# Genomic Androgen Receptor-Occupied Regions with Different Functions, Defined by Histone Acetylation, Coregulators and Transcriptional Capacity

**DOI:** 10.1371/journal.pone.0003645

**Published:** 2008-11-10

**Authors:** Li Jia, Benjamin P. Berman, Unnati Jariwala, Xiting Yan, Jon P. Cogan, Allison Walters, Ting Chen, Grant Buchanan, Baruch Frenkel, Gerhard A. Coetzee

**Affiliations:** 1 Department of Preventive Medicine, University of Southern California, Los Angeles, California, United States of America; 2 Department of Urology, University of Southern California, Los Angeles, California, United States of America; 3 Department of Biochemistry and Molecular Biology, University of Southern California, Los Angeles, California, United States of America; 4 Department of Orthopedic Surgery, University of Southern California, Los Angeles, California, United States of America; 5 Norris Cancer Center, University of Southern California, Los Angeles, California, United States of America; 6 Institute of Genetic Medicine, University of Southern California, Los Angeles, California, United States of America; 7 Department of Biological Sciences, University of Southern California, Los Angeles, California, United States of America; 8 Epigenome Center, Keck School of Medicine, Molecular and Computational Biology Program, University of Southern California, Los Angeles, California, United States of America; National Institute on Aging, United States of America

## Abstract

**Background:**

The androgen receptor (AR) is a steroid-activated transcription factor that binds at specific DNA locations and plays a key role in the etiology of prostate cancer. While numerous studies have identified a clear connection between AR binding and expression of target genes for a limited number of loci, high-throughput elucidation of these sites allows for a deeper understanding of the complexities of this process.

**Methodology/Principal Findings:**

We have mapped 189 AR occupied regions (ARORs) and 1,388 histone H3 acetylation (AcH3) loci to a 3% continuous stretch of human genomic DNA using chromatin immunoprecipitation (ChIP) microarray analysis. Of 62 highly reproducible ARORs, 32 (52%) were also marked by AcH3. While the number of ARORs detected in prostate cancer cells exceeded the number of nearby DHT-responsive genes, the AcH3 mark defined a subclass of ARORs much more highly associated with such genes – 12% of the genes flanking AcH3+ARORs were DHT-responsive, compared to only 1% of genes flanking AcH3−ARORs. Most ARORs contained enhancer activities as detected in luciferase reporter assays. Analysis of the AROR sequences, followed by site-directed ChIP, identified binding sites for AR transcriptional coregulators FoxA1, CEBPβ, NFI and GATA2, which had diverse effects on endogenous AR target gene expression levels in siRNA knockout experiments.

**Conclusions/Significance:**

We suggest that only some ARORs function under the given physiological conditions, utilizing diverse mechanisms. This diversity points to differential regulation of gene expression by the same transcription factor related to the chromatin structure.

## Introduction

The ‘textbook’ paradigm of gene regulation by steroid hormone receptors entails the binding of receptors to hormone response elements located 5′-upstream of the transcription start sites (TSSs) of responsive genes, followed by the recruitment of non-DNA-binding coactivators or corepressors. These latter factors modify histones and interact with the basal transcriptional machinery to modulate transcriptional initiation [1]. This paradigm is based on, and has been the basis for, many studies in which steroid hormone receptor binding motifs, 5′-upstream of TSSs of target genes, were identified and characterized. Thus, DNA binding locations for steroid receptors were found where investigators looked for them. More recent data however have revealed that the distribution of steroid receptor occupancy genome-wide has no preference for 5′-flanking sequences of annotated genes [2], [3]. Moreover, the distribution of such sites, including androgen receptor (AR)-occupied regions (ARORs) on chromosomes 21 & 22 [3], is poorly correlated with gene density. For this reason, two studies that used genomic windows around the TSSs of annotated genes may have been limited in their capacity to assign ARORs in a genome-wide fashion [4], [5] by missing functional ARORs far away on linear DNA. Nonetheless, it is not a trivial task to assign *a priori* functionality to ARORs if they are not directly associated with promoters of nearby genes. One potential way to better assign functionality to transcription factor binding sites, including ARORs, is to make use of chromatin analyses. In particular, ARORs containing active histone modifications may function as enhancers, modulating transcription at a distance.

Histone covalent modifications, also referred to as chromatin epigenetics, are involved in normal somatic cell development as well as in the progression of diseases, such as cancer [6], [7]. Such histone modifications or ‘marks’ lead to the recruitment and subsequent docking of regulatory protein complexes that modulate transcription. These marks provide a nuanced chromatin ‘language’ that demarcate chromatin into structural domains with dynamic functional consequences [8]. Systematic studies of chromatin modifications have revealed a complex landscape including ‘punctate’ sites of modified histones (histone H3 lysine 9/14 acetylation and lysine 4 di- and trimethylation) at transcription start sites and distal regulatory elements [9], [10]. Histone H3 acetylation is an epigenetic mark that allows prediction of functional regulatory elements [11]. The molecular mechanisms of how chromatin remodeling modulates transcription in the context of many diseases, including prostate cancer (PCa), will undoubtedly have a major impact in disease understanding and management. In the case of PCa, it is known that the progression to the fatal stage of the disease, ablation resistance, depends to a large degree on the activity of the AR [12]–[16], a potent transcription factor (TF) that facilitates epigenetic control of gene expression at many target loci across the entire human genome.

With the development of high throughput methodologies such as ChIP-chip (reviewed in [17]) and more recently ChIP-seq (e.g. [18], [19]), it has become possible to comprehensively map regions in the genome of mammalian cells that are occupied by TFs of interest. Often, such studies identify many regions that are occupied without any apparent functional consequence [20], [21]. To determine genomic AR binding sites and better understand their biological significance, we mapped both AR-occupied regions (ARORs) as well as histone H3 acetylation (AcH3) states chromosome-wide (large parts of chromosomes 19 and 20) in the C4-2B aggressive PCa cell line. We chose the AcH3 mark, as it is a well-characterized marker of active enhancers and promoters [10], [22]. Of the ARORs identified, only a subset has clear functional consequences in the PCa cell line analyzed, indicating the existence of diverse AR functions under changing physiological circumstances.

## Results

### AR-occupied regions in prostate cancer cells exceed number of DHT-responsive genes

**Figure 1:**
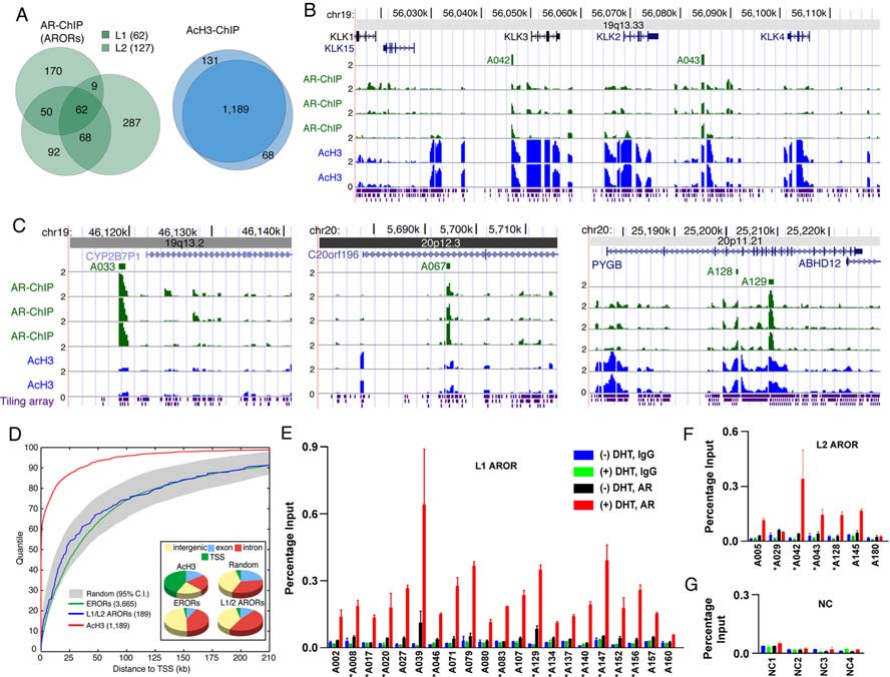
Characterization of ARORs and AcH3 regions on chromosome 19 and 20. Three replicate ChIP-chip experiments identified 738 Androgen Receptor Occupied Regions (ARORs), 62 of which were common to all three replicates (L1 ARORs) and 127 common to only two of three (L2 ARORs), while two replicate ChIP-chip experiments identified 1,388 regions with acetylated histone H3 marks, 1,189 of which were present in both replicates (A). Genome plots are shown for the kallikrein locus (B) and three other AROR-containing loci (C), where AR-ChIP peaks are labeled, and raw log2 ratios [from 0 (1-fold) to 2 (4-fold)] for each replicate are shown in green (AR-ChIP) and blue (AcH3-ChIP). Panel (D) shows the genomic positioning of the 189 L1/L2 AROR peaks and 1,189 AcH3 peaks. A cumulative distribution plot (outer) shows that the distance from annotated transcription start sites (TSSs) is similar between ARORs, Estrogen Receptor Occupied Regions (ERORs) from [2], and randomly selected regions from the repeat-masked tiling array, while a majority of AcH3 peaks are located at or near TSSs. All three classes (AROR, EROR, and AcH3) are excluded from exons relative to randomly selected regions (insert). The selected ARORs were validated by independent ChIP-qPCR (E–G). C4-2B cell were incubated in phenol red-free RPMI 1640 containing 5% CSS for 3 days and then treated with 10 nM DHT or ethanol (EtOH) vehicle for 4 h. Conventional site-specific ChIP assays were performed with anti-AR antibody. Normal IgG was used in parallel. Twenty-one L1 ARORs (E), 7 L2 ARORs (F) and 4 negative control (NC) (see table S4) (G) regions were examined by TaqMan qPCR. Acetylated ARORs are indicated by asterisks. A042 (F) is the PSA enhancer and acted as a positive control. All values are presented as percentage of input.

We performed three independent AR ChIP-chip analyses using a genomic tiling microarray (NimbleGen Systems Inc., Madison, WI) that covers sequences on chromosomes 19 and 20 that total about 3% of the human genome, including the classical AR target gene *PSA/KLK3*. We implemented a novel peak-calling algorithm that allowed us to identify 189 genomic regions (about 500 bp on average) occupied by the AR in at least two of the three experiments, with 62 [level 1 (L1) ARORs] being reproduced in all three, and 127 [level 2 (L2) ARORs] being reproduced in exactly two of the three (Figure 1A). Their coordinates are provided in Table S1, where they are numbered consecutively from A001 to A189. The AROR peaks were generally robust in multiple sampling experiments (see Materials and Methods). Frequently, ARORs were classified as L2 and not L1 because of missing probes imposed by repetitive sequences and requirements of the NimbleGen design criteria. For instance, AROR A042 encompasses the well-characterized *PSA/KLK3* enhancer [23]–[25], but was called as an L2 AROR primarily because it partly overlaps a repeat-masked region (Figure 1B). A043 identifies a novel AROR at the KLK locus (Figure 1B), and four other examples, ARORs A033, A067, A128 and A129, are displayed in Figure 1C. The AROR calling in our ChIP-chip analysis has a very low false positive discovery rate, which is reflected by the fact that 21/21 L1 ARORs (Figure 1E) and 6/7 L2 ARORs (Figure 1F) could be validated by site-specific independent ChIP assays. For the four negative control (NC) regions, two of which (NC1 and NC2) contain canonical androgen response elements (ARE), no AR occupancy was detected (Figure 1F). Importantly, with the exceptions of A039 and A129 (Figure 1E), we found little evidence of ligand-independent AR occupancy, which is in line with previous observations at the canonical AR responsive enhancer for the *PSA* gene [24], [26]. These exceptions, however, may have important implications for ligand-independent signaling of the AR as part of the ablation-resistant phenotype in advanced prostate tumors.

Two replicates for the AcH3 ChIP-chip experiment were sufficient because they were highly reproducible – of 1,388 regions identified within the two replicates, 1,189 (>90%) were in common (Figure 1A). This high reproducibility is attributable to the stability of this lineage-specific histone modification. Mapping of the AcH3 peaks to the human genome show that 48% of them overlap with TSSs (Figure 1D, insert). In contrast, very few ARORs coincide with TSSs, indicating that AR rarely occupies proximal promoters. In fact our ARORs, like the ER-occupied regions (ERORs) observed in MCF7 breast cancer cells [2], were distributed randomly with respect to distance from TSSs (Figure 1D) and were thus mainly located in introns or intergenic sequences (Figure 1D, insert). This distribution was very similar to that obtained for ARORs in chromosomes 21 & 22 of LNCaP cells (Figure S1) [3]. In terms of evolutionary conservation, both the ARORs and ERORs were only slightly more conserved across evolution than a set of random control sequences that exclude repetitive sequences not represented on the tiling array (Figure S2).

**Figure 2:**
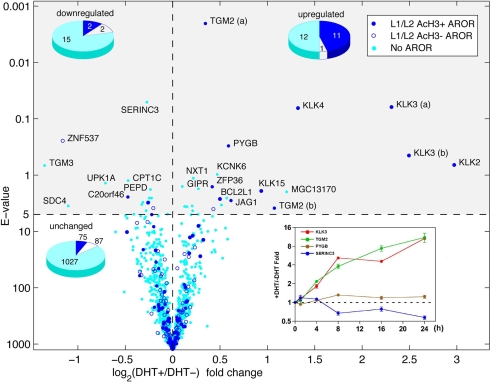
DHT-responsive genes in C4-2B cells. Illumina expression arrays were used to measure expression levels of 46,713 transcripts in three replicates before and three replicates after DHT exposure in C4-2B cells, including the 1,232 RefSeq transcripts within our chromosome 19/20 genome tiling arrays, which are shown here. The student's t-test was used to determine statistical significance, and p-values were adjusted based on random permutations of the full dataset. This volcano plot shows the E-value (number of transcripts at the given p-value expected by chance) plotted against the mean fold change. 24 transcripts up-regulated at the E = 5 level (permutation-adjusted p-value = 0.003) and 19 transcripts down-regulated, are shown in the upper two quadrants. Transcripts are color-coded based on whether they are adjacent to an AROR, and the up-regulated transcripts show an elevated number adjacent to acetylated, but not un-acetylated, ARORs. The inset shows a time course of endogenous gene expression. C4-2B cells were cultured in hormone-depleted medium for 3 days and then treated with DHT (10 nM) or ethanol vehicle for the indicated times. Expression levels of 4 representative genes were measured by real-time RT-PCR. The data is normalized to 18S expression in log scale; Values are fold changes over the vehicle control at each time point.

Extrapolation of our chromosome 19/20 results suggests that under similar experimental and computational stringency we would expect in the human genome just over 2,000 ARORs that would be classified as L1 and around 6,300 as L2. Interestingly, these estimates well exceeded the number of DHT-responsive genes in C4-2B cells as assessed using genome-wide gene Illumina expression arrays (primary data presented in Table S7). Even when responsiveness was conservatively defined using a t-test statistical Expectation Value (E-value) cutoff of 150 (permutation-adjusted p-value = 0.003), only 552 of 46,713 transcripts (1.2%) were identified as stimulated and 416 (0.9%) repressed by DHT at 16 hours compared to vehicle control (Figure S3 and Table S2). When we confined the analysis to the 1,232 RefSeq transcripts corresponding to the ChIP-chip analyzed areas of chromosomes 19/20, only 24 (1.9%) were stimulated and 19 (1.5%) repressed by DHT at the E<5 (permutation-adjusted p-value = 0.004) level (Figure 2). Yet, of the 178 transcripts containing or adjacent to an L1 or L2 AROR, only 16 (9.0%) were regulated by DHT (12 stimulated, 4 repressed). Quantitative RT-PCR time-course analyses of four of these genes (*KLK3*, *PYGB*, *TGM2* and *SERINC3*) (Figure 2 insert) verified both the Illumina expression data and the choice of the 16-hour time point after DHT treatment. As such, it appears that the majority of ARORs have no nearby genes responsive to DHT in these prostate cancer cells, begging the question of their functionality.

### Histone H3 Acetylation defines a subclass of ARORs more highly associated with DHT-stimulated genes

**Figure 3:**
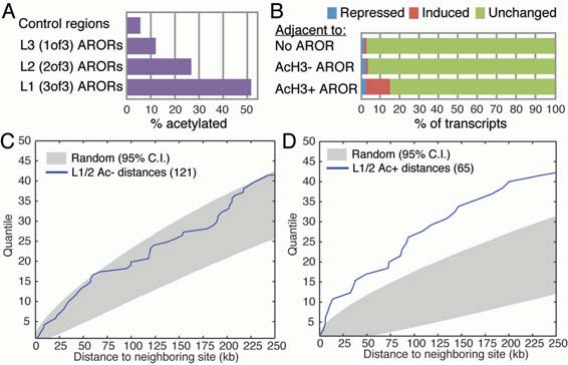
Histone H3 acetylation defines a distinct subclass of ARORs. Histone H3 acetylation peaks overlap 52% of the most reproducible L1 ARORs, but only 27% of the L2 ARORs, 12% of the L3 ARORs, and 6% of random sequences from the area covered by the chromosome 19/20 tiling array (A). Transcripts adjacent to acetylated ARORs are significantly more likely to be up-regulated in C4-2B cells, with 12.5% of transcripts showing up-regulation, as opposed to 1.1% of those adjacent to un-acetylated ARORs, and 1.1% of those not adjacent to any AROR; this was not the case for repressed genes (B). While un-acetylated L1/L2 ARORs are about as likely as randomized controls to cluster on the genome as shown by the cumulative distribution plot of inter-AROR distances (C), acetylated ARORs show significantly more genomic clustering (D).

AR occupancy at many regions identified in our study does not lead to alterations in expression of nearby genes as a function of DHT exposure. Since AcH3 is a landmark of functional enhancers [10], we examined the AcH3 state of the ARORs to determine their possible functionality. Critically, 52% of L1 ARORs, 27% of L2 ARORs and 12% of L3 ARORs (ARORs detected in only 1 of 3 ChIP-chip experiments) overlapped with AcH3 peaks, which is progressively in excess of the 6% overlap predicted for random association (Figure 3A).

To determine whether AROR acetylation has any power to predict DHT responsiveness of nearby genes, we compared the frequency of stimulated, repressed, and unaffected genes at loci that contain an acetylated AROR(s), non-acetylated AROR(s), or no AROR at all. The results (Figure 3B) show that AcH3+ARORs are relatively enriched for DHT-stimulated transcripts. Specifically, 12.5% of the genes flanking AcH3+ARORs were DHT-stimulated (note that the expected maximum is less than 100%, as we don't necessarily expect an intergenic AROR, even if functional, to target transcripts *both* upstream and downstream from the AROR). This is highly enriched (p = 1.4×10^−7^ by Fisher exact test) relative to AcH3- ARORs and control regions without an AROR, for both of which only 1.1% of adjacent genes were DHT-stimulated. There was no statistically significant relationship between repressed genes and association with ARORs, irrespective of their acetylation status. Conversely, 11 of 24 up-regulated genes were adjacent to an acetylated AROR, while only 1 of 24 was adjacent to an un-acetylated AROR (Figure 2). While these results demonstrate a strong correlation between the acetylation of an AROR and DHT-mediated stimulation of nearby gene(s), the majority of genes located in close proximity to ARORs, even acetylated ones, were unresponsive (Figure 3B).

We additionally found that acetylated ARORs (Figure 3D) were more likely to occur in clusters than were non-acetylated ARORs (Figure 3C) (e.g. *PYGB* AROR cluster in Figure 1A). This is consistent with a transcriptional role for all the acetylated ARORs, since enhancer elements with established functions are often clustered to assure fidelity of transcriptional enhancement by providing redundancy or synergy among individual elements [27].

### Most ARORs contain potential enhancer activity

**Figure 4:**
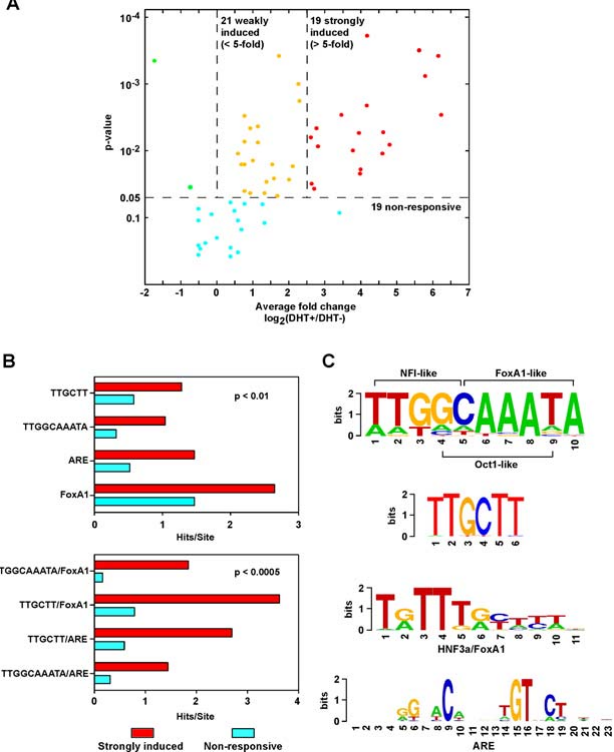
Most ARORs have enhancer potential (transactivation activity), which is associated with several sequence motifs. Transient transfection luciferase reporter assays were carried out in duplicate, and repeated independently at least 3 times for 61 of 62 L1 ARORs before and after DHT exposure, and the results are displayed in a volcano plot (4A) which shows the fold change between DHT+ and DHT- luciferase levels and the p-value significance level by the Student's t-test. At the p<0.05 level, 19 ARORs (31%) were strongly induced, while 21 ARORs (34%) were weakly induced. From L1 ARORs, 52 sequence motifs from Transfac and *de novo* motif discovery algorithms were found to be significantly enriched (see text). We performed chi-square tests to determine those motifs (B, upper) and motif pairs (B, lower) significantly enriched in the 18 strongly induced (red bars) vs. the 19 non-responsive (blue bars) ARORs. Four motifs were identified (C), including binding sites for FoxA1, the AR, along with two de novo motifs with similarity to FoxA1, NFI, and Oct1.

We suspected that in many of the ARORs in C4-2B cells, the AR was present in complexes devoid of transcriptional trans-activation activity. We therefore tested the DHT-mediated enhancer activity of 61 ARORs in transient transfection reporter assays. These 61 ARORs, encompassing all of the highly reproducible (L1) ARORs except A059 (which is in the middle of a simple tandem repeat), were cloned upstream of a thymidine kinase (*TK*) minimal promoter/luciferase reporter and subjected to luciferase assay in the presence or absence of DHT exposure as described in Materials and Methods. As shown in Figure 4A, forty of the 61 L1 ARORs (66%) displayed DHT-dependent enhancer activity with p<0.05 (single tailed t-test). Of these, 19 ARORs stimulated activity of the heterologous TK promoter by >5-fold. Thus, most of the ARORs have intrinsic enhancer potential, although in most cases this potential is either not materialized or not detectable when the AROR is assessed in its native context based on responsiveness of adjacent genes in C4-2B cells (Figure 2 and see Discussion). Even in the luciferase assays, where the ARORs are tested in a relatively promiscuous environment, we observed significant variability in enhancer activity (Figure 4A), which was not attributable to the level of occupancy (Figure 1E).

Combinatorial regulation is a fundamental and an increasingly central aspect of transcriptional control in higher eukaryotic cells, and we therefore hypothesized that (*i*) the ARORs might be enriched for binding sites for both AR as well as AR-coregulators, and (*ii*) the presence of such sites modulated responsiveness to DHT in a combinatorial manner. To pursue the *first hypothesis*, we searched for TF binding motifs enriched in the highly reproducible L1 ARORs compared to 6200 equivalent random size-matched sequences from the interrogated genomic territory on chromosomes 19 and 20. These analyses excluded the un-cloned AROR A059, as well as A002 that contains 14 tandem canonical AREs and would skew the analysis. The set of 60 L1 ARORs were investigated using both scanning and *de novo* motif-finding approaches. Scanning approaches using the Cis-Elements Annotation System (CEAS) website [28] to map potential binding sites of the 550 vertebrate TF binding motifs represented in the TRANSFAC database [29], identified five potential TF binding sites highly over-represented within the ARORs. These were ARE (3.6-fold enrichment, p = 7.2×10^−12^), HNF3α/FoxA1-binding motif (1.8-fold, p = 2.2×10^−16^), NFI-binding motif (1.9-fold, p = 2.7×10^−9^), GRE (1.5-fold, p = 2.5×10^−12^), and C/EBP (1.4 fold, p = 4.3×10^−13^) (Table S3). Nonetheless, only 10 of 60 L1 ARORs (17%) contained AREs that could be considered high stringency (PWM log-odds>8.5), whereas 38 of 60 (63%) contained AREs with low stringency (PWM log-odds>5.0). *De novo* approaches utilizing BioProspector [30], MDscan [31] and Weeder algorithms [32] yielded a total of 525 recurring motifs within the 60 L1 ARORs. To collapse the many similar motifs into similarity groups, we employed linear hierarchical clustering based on highly overlapping motifs (see Materials and Methods). This analysis resulted in 45 motifs (Table S3), of which three (ARE, GRE and FoxA1) had been found by CEAS as detailed above.

We next compared the distribution of the 50 motifs from both scanning and *de novo* motif-finding approaches, as well as two alternate ARE versions from the ConSite [33], between 18 ARORs (A002 was excluded from the 19 strongly induced ARORs, not to skew the data) most responsive to DHT in luciferase enhancer assays with the 19 least responsive (Figure 4A). Four motifs were significantly enriched (p<0.01, t-test) in the strongly responsive ARORs (Figure 4B): ARE (2.2-fold), FoxA1 (1.8-fold), and two *de novo* motifs with consensus sequences of TTGCTT (2.2-fold) and TTGGCAAATA (3.2-fold) (Figure 4B and C, Figure S4). The TTGGCAAATA motif appears to resemble overlapping binding motifs for NFI, FoxA1 and Oct-1 (Figure 4C).

Pair-wise analysis of the 50 motifs and the two alternate AREs identified only four random pair combinations significantly enriched in the 19 most responsive ARORs (p<0.0005) (Figure 4B). Two of these ‘response motif-pairs’ consisted of an ARE with either the TTGCTT (4.6-fold enrichment) or TTGGCAAATA (4.6-fold) *de novo* motifs. The remaining two did not have AREs, but instead consisted of a FoxA1-binding site with either TTGCTT (4.6-fold) or the TTGGCAAATA (11.3-fold) motifs (Figure 4C).

### Combinatorial regulation by AR coregulators

**Figure 5:**
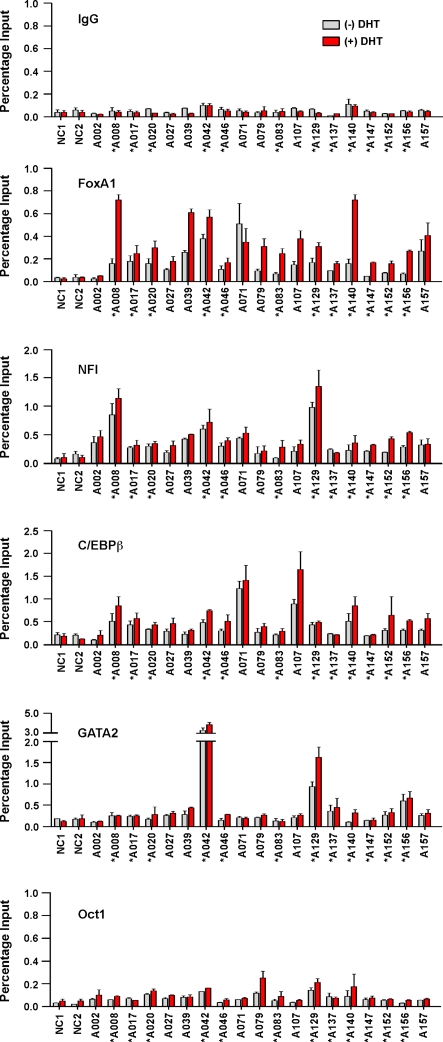
Occupancies of AR-coregulators on selected ARORs. C4-2B cells were cultured in hormone-depleted medium for 3 days and then treated with 10 nM DHT or ethanol vehicle for 4 h. Conventional site-specific ChIP assays were performed with indicated antibodies. Nineteen ARORs and 2 negative control (NC) regions were examined by qPCR. The values are presented as percentage of input.

The identification of motifs enriched in ARORs (Table S3), and in particular in responsive ARORs (Figure 4B and C), prompted us to investigate the role of FoxA1, C/EBP, NFI and Oct-1 in modulating AR genomic action, as well as the role of GATA2, which has been recently implicated in AR genomic action in LNCaP cells [3]. We tested whether these TFs are constitutively present at ARORs in C4-2B cells, and whether occupancy is affected by DHT-driven AR binding as tested in Figure 1E. ChIP assays were performed with the respective antibodies, and occupancy was determined at the 18 L1 ARORs tested in Figure 1E, as well as the A042 L2 AROR from the known AR target *PSA/KLK3* locus. As shown in Figure 5, occupancy at the basal state and in response to DHT varied significantly as a function of both the AROR and the transcription factor of interest. For example, FoxA1 was present at almost every AROR with occupancy in most cases increasing following treatment with DHT. Notable exceptions included the lack of FoxA1 recruitment to AROR A002 (which contains 14 AREs), and the inability of DHT to enhance FoxA1 occupancy of AROR A071. NFI and C/EBPβ occupancy also varied considerably among ARORs, but was less responsive to DHT. C/EBPα in contrast, which has been found at ER-occupied regions in MCF7 breast cancer cells [2], was not detected at any of our ARORs in C4-2B prostate cancer cells (data not shown). GATA2 occupied only acetylated ARORs near three DHT-responsive genes; *PSA* (A042), *PYGB* (A129) and *TGM2* (A156), and Oct-1 interaction was weakly observed on several ARORs with only one of three different antibody preparations. Interestingly, we could not always predict occupancy of any particular AROR by FoxA1, C/EBP, NFI and Oct-1 by the presence or absence of consensus motifs. This finding is suggestive of complex protein-protein interactions in the recruitment of a given TF to a specific AROR, and further indicates that the site-specific arrangement of TFs creates a diversity of site-specific AR-mediated transcriptional responses. For example, A129 exhibited relatively high NFI and GATA2 occupancy, but low to intermediate C/EBPβ and FoxA1 levels, A107 had relatively high C/EBPβ, low to intermediate NFI and FoxA1 and negligible GATA2, whereas A140, was highly occupied by FoxA1 after DHT treatment but had low NFI, intermediate C/EBPβ and extremely low GATA2 occupancy levels.

The C4-2B cell line was originally obtained from LNCaP cells grown as xenografts after castration [34]. They are therefore considered ‘androgen-independent’ although AR-mediated gene expression is still quite responsive to androgen treatments [24]. We compared C4-2B with LNCaP cells with respect to AR and TF occupancies (Figure S6). The occupancy levels in the absence and presence of DHT were similar between the two cell lines, indicating that the AR and other TF occupancies are not dramatically affected by the androgen-dependent status of the cells.

**Figure 6:**
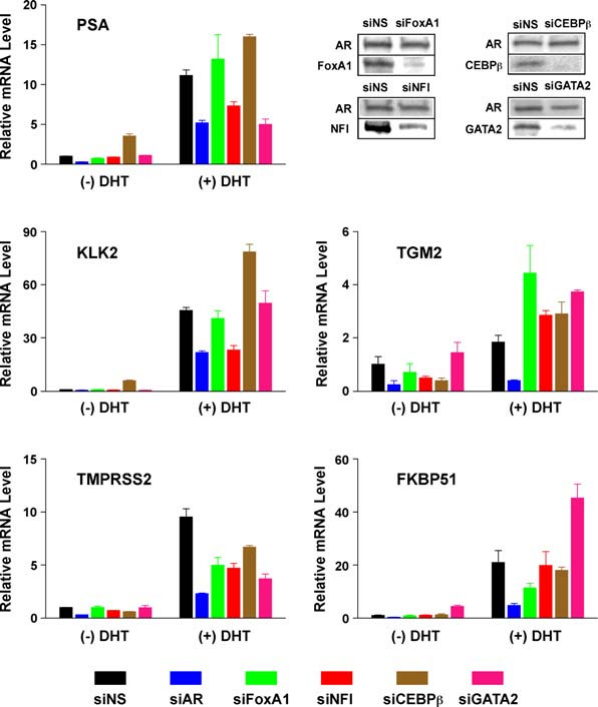
Expression of AR target genes after siRNA-mediated knockdown of coregulators. C4-2B cells were transfected with siRNA targeting AR, FoxA1, NFI, CEBPβ, GATA2 or non-specific (NS) regions. Two days after transfection, cells were treated with 10 nM DHT or ethanol vehicle for 18 h. Expression levels of five AR target genes were examined by real-time RT-PCR. Relative mRNA level was normalized by *GAPDH* mRNA. Efficiency of each siRNA knockdown was measured by immunoblot with indicated antibodies (upper right).

Using siRNAs to knock down the levels of endogenous FoxA1, C/EBP, NFI and GATA2, we investigated the role of each of these transcription factors in DHT-stimulation of AR target genes. Remarkably, DHT responsiveness varied with both the TF investigated and amongst the five tested AR target genes [*PSA/KLK3* (near A042), *KLK2* (near A043), *TGM2* (near A156), *TMPRSS2* (chromosome 21) and *FKBP51* (chromosome 6)] (Figure 6) in a manner not attributable to differential occupancy (compare Figures 5 and 6). For example, knockdown of FoxA1 decreased DHT-responsiveness of *TMPRSS2* and *FKBP51*, enhanced that of *TGM2* and did not affect *PSA* and *KLK2* expression. In contrast, knockdown of NFI decreased DHT-responsiveness of *PSA*, *KLK2* and *TMPRSS2*, increased that of *TGM2*, and did not affect *FKBP51*. Knockdown of GATA2 sensitized *TGM2* and *FKBP51* to DHT induction. Interestingly, knockdown of C/EBPβ strongly increased basal expression of *PSA* and *KLK2*, which may reflect modification of AR action on these genes in the absence of added ligand [24]. We had previously analyzed occupancy at ARORs close to PSA (A042) and TGM (A156) (Figure 5); it is interesting to note that occupancy by FoxA1, NFI, C/EBPβ, and GATA2 were all present at the ARORs close to the genes (re-plotted as Figure S7), and likely exerted their actions via these sites. The diverse effects of each of the coregulators on AR function may therefore reflect a complex regulatory mechanism suitable for modulation of DHT responsiveness under different physiological conditions.

## Discussion

The functional consequences of AR engagement at DNA target sites, which normally follow androgen binding to the AR, are of major importance in the development of the male phenotype and in diseases such as PCa. The prostate is a well-established AR target tissue, where it is known that 5α-reductase converts circulating testosterone to the more potent androgen, DHT. DHT in turn binds the AR, mediates its nuclear translocation, and promotes prostate differentiation via specific gene expression modulation. With respect to PCa, increased life-time exposure to enhanced AR activity predisposes men to the disease [13]. Even more striking, aberrant AR activity seems to be necessary and sufficient to convert androgen-dependent prostate tumors to ablation resistant ones; this fact has made the AR an attractive target for PCa therapy [16], [35], [36]. Although non-genomic actions of the AR have been proposed [37], its major activity is thought to be a consequence of DNA engagement and transcriptional regulation. Due to progress in genomic techniques measuring engagement of TFs, the quantitative and qualitative mapping of AR/DNA engagement profiles have become the basis of a limited number of studies during the past year [3]–[5], however, the control of such engagement and its consequences remain relatively unexplored.

In this report, we employed ChIP-chip to map the locations of both the AR and AcH3 onto 3% of the genome in C4-2B prostate cancer cells. AR occupancy exhibited higher inter-experimental variance than AcH3, but was still sufficiently reproducible to detect 62 regions occupied in every one of three independent experiments. Aside from their exclusion from exonic sequences, ARORs were distributed randomly with respect to gene locations. Of the 62 highly reproducible ARORs, 32 were also marked by AcH3. The significance of AROR acetylation was apparent when examining gene expression patterns of neighboring genes – 12% of genes adjacent to AcH3+ARORs were DHT-stimulated, compared to only 1% of genes adjacent to AcH3- ARORs (conversely, 46% of up-regulated transcripts were adjacent to acetylated ARORs whereas only 4% were adjacent to an un-acetylated AROR.) While the number of differentially regulated genes on chromosomes 19 and 20 is small, this roughly 10-fold enrichment in functionality of ARORs with the acetylation mark illustrates the utility of combining ChIP studies of individual transcription factors with epigenetic markers in the same cells.

**Figure 7:**
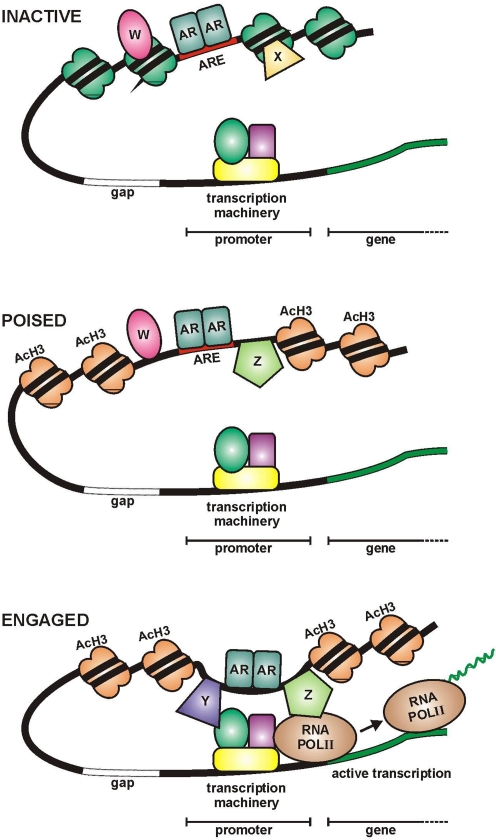
Schematic model of AROR functions. Three types of AR/DNA engagements are envisioned. Inactive ARORs represent AR occupying sites of relatively condensed chromatin along with coregulators W & X; these sites may simply be reservoir of AR to be used during dramatic changes in physiological conditions. Poised ARORs represent AR at AcH3 modified sites ready to engage the transcription initiation machinery, but held in check by coregulaters W & Z. Engaged ARORs represent AR actively mediating transcriptional control of target gene(s) by looping across varying genomic distances perhaps assisted by coregulators Y & Z.

We detected many more ARORs than DHT-responsive genes, leaving most ARORs without any detectable function in C4-2B cells. We can speculate that these ARORs, particularly the acetylated ones, modulate transcription at levels undetectable in our assay, or target microRNAs or other un-annotated transcripts. They may even function as enhancers of genes over large linear DNA distances or on other chromosomes [38]. Transcriptional enhancement of other genes could have been offset by compensatory mechanisms of RNA destabilization, or difficult to detect due to high basal steady state levels. Finally, it is plausible that occupancy at some ARORs was without any transcriptional function under the experimental conditions employed. Some of these ARORs, in particular acetylated ARORs, could be poised for transcriptional engagement upon arrival of a missing signal. Others may have functions unrelated to transcription, such as chromosome structural regulation, replication or DNA repair [21]. Still others, especially non-acetylated ARORs, may have no function at all. Thus, we envision many (even most) ARORs are inactive in relatively condensed chromatin at any given time and situation with or without coregulators (Figure 7, W & X); the latter my act as pioneers to facilitate the original recruitment of AR to the site. Some ARORs may be poised to act because they contain modified chromatin (AcH3) and a different complement of coregulators (Figure 7, W & Z). Only a small number of ARORs is actually engaged in transcriptional regulation possibly again due to the presence of another set of coregulators (Y & Z) that may convert poised ARORs to engaged ones. We propose that engaged ARORs will differ in activity depending on the physiological and temporal states of the cells. W, X, Y & Z in figure 7 represent many combinations of coregulators acting in a site-specific manner allowing for qualitative adjustment of AR signaling under different situations.

Transcriptional enhancer activity of ARORs in a standard luciferase assay of a minimal TK promoter demonstrated that 66% of the L1 ARORs, including many without apparent influence on endogenous genes, had transcriptional enhancement potential in *in vitro* assays. Indeed, even ARORs that had no enhancer activity in C4-2B cells turned out to be active in other prostate cancer cell lines (data not shown). As stated above, whether an AROR is engaged, poised, or transcriptionally inactive, even in a non-chromosomal context after transient transfection, likely depends on the differential occupancy of the AROR by trans-acting factors, which modify the transcriptional activity of the AR. This was best exemplified by the siRNA knockdown of specific AR coregulators that had differential effects on different DHT-responsive genes (Figure 6). These AR coregulators, including FoxA1, C/EBPß, NFI and GATA2, were identified here and/or in other studies [3].

FoxA1 and C/EBPα have both been implicated previously in estrogen receptor (ER)-mediated [2] and FoxA1 in AR-mediated [3] gene expression. The Matusik lab further showed that FoxA proteins were expressed in the prostate and interacted with the AR, thus modulating its activity on some but not all promoters [39]. Although FoxA2 may play a role in prostate cancer progression to androgen independence [40], it is not expressed in C4-2B cells (data not shown). FoxA transcription factors may act as pioneer factors, due to their winged-helix structures, and may bind to chromatin predominantly at distant enhancers as an initial event to which subsequent TF may be recruited. Their binding seems to be dependent on histone H3K4 methylation and they likely establish lineage-specific transcription [41]. Our data, on the other hand, have shown that in addition to FoxA1 being attracted to certain ARORs in the absence of hormone, at other sites FoxA1 recruitment was increased after DHT treatment indicating that FoxA1 in PCa cells has additional AR-dependent functions.

C/EBPs (CCAAT-enhancer-binding proteins) belong to a family of transcription factors that interact with the CCAAT box motif, which is present in the promoters of several genes. In LNCaP cells, C/EBPα over-expression inhibited the expression of PSA [42], indicating that it is a possible AR negative regulator. However, C/EBPβ seems more relevant in cancer cell control in a positive way, since it is hormonally controlled and involved in cell proliferation, whereas C/EBPα is predominantly expressed in the terminal differentiation phases of epithelial cells. For example, C/EBPβ appears to be a key transcription factor in cell growth, since its depletion by RNA interference blocked the stimulation of growth hormone [43]. In our study C/EBPβ (and not C/EBPα) was found at many ARORs.

Nuclear factor I (NFI) belongs to a family of transcription factors that play important roles in modulating transcription of differentiation-associated genes [44]. Interestingly, a two-step synergistic model was proposed to explain the interaction and activity of progesterone receptor and NFI [45]. Although our siRNA knockdown experiments indicated diverse effects of NFI on gene regulation, specific roles for NFI in AR signaling have not yet been proposed, and we believe our findings are novel and may well reveal an important pathway in AR signaling and PCa progression.

GATA family members are able to bind to DNA at their response elements and mediate the ‘opening’ of compacted chromatin [46] and have been proposed to play major roles in endocrine function and disease [47]. Furthermore, GATA2 was previously found as an AR coregulator at AR sites on chromosome 21 and 22 [3]. However, in our study GATA2 was found only at three AROR sites. Interestingly, all three were in the proximity of genes that were substantially stimulated by DHT treatment (*PSA*, *TGM2* and *PYGB*), indicating that this transcriptional coregulator might potentiate the AR-mediated transcription of these genes. However, depending on the specific enhancer, GATA2 might have enhancing or repressing activities on gene expression, as was shown in the siRNA knockdown experiments.

Oct-1 was proposed as an AR collaborator [3] and was shown to physically interact with the AR in a DNA-dependent manner that facilitated cofactor recruitment [48], [49]. However, we found only very low levels of Oct-1 at some ARORs, indicating that the participation of Oct-1 is limited to perhaps only very few AR regulated regions where it may play a specialized function.

We have previously reported that at some loci (but not at others) gene-wide histone H3 acetylation leads to efficient AR-mediated transcription in C4-2B but not in LNCaP cells [24]. Apart from the different kallikrein loci (Figure 1B), we did not find any other gene-wide acetylation profiles on chromosomes 19 & 20, indicating that the expression of kallikreins is perhaps examples of only a few genes marked in this way. From the work presented here it is clear that coregulators also contribute significantly to transcriptional efficiency.

The interplay between the AR and potential coregulators is very likely promoter/enhancer specific and dependent on the phenotypes of the target genes as was shown in the present study. Some of the AR coregulators may be considered pioneer factors, laying the groundwork to allow efficient AR-mediated gene modulation as was proposed for ER activity [2]. In such cases they may even provide the initial site for the AR to dock at enhancers or promoters that do not contain strong AREs; in this scenario, the AR is brought into play via its interaction with the coregulator, with weak binding to, or even no contact with DNA. A complex site-specific matrix of transcription factors may therefore exist to allow specific gene regulation under specific physiological conditions. At this stage we do not know the rules governing AR-mediated expression control at different loci. As a matter of fact, there are likely dozens more coregulators that determine AR activity across all ARORs and cell types. Deciphering the rules of AR engagement by these coregulators across various ARORs and under different physiological and pathological conditions may require decades of research. Meanwhile, measuring epigenetic marks, such as histone H3 acetylation, provides a powerful approach as a functional adjunct to characterize ARORs. The combined mapping of TF-binding regions and histone modifications may therefore prove a beneficial principle to be used for the functional, genome-wide characterization of not only AR, but also that of many other TFs.

### Conclusions

Over the past several decades a general concept of TF-mediated gene regulation has emerged. It depicts a sequence of events, starting with TFs binding at specific regions of DNA, followed by the covalent modification of chromatin proteins (primarily histones) to ‘open’ chromatin, and ultimately allowing the nearby docking of the transcription machinery. This is not necessarily always the case and alternative insights were recently revealed by the use of new high throughput techniques, which allow the agnostic mapping of such regions and an unbiased appreciation of their functions. In the current study, many AR binding sites were not associated with nearby gene expression despite the fact that they had intrinsic positive regulatory activity in chromatin-independent assays. On the other hand, those that regulated the expression of nearby genes were 10-times more likely marked by acetylation, indicating that AR occupancy and associated histone acetylation are both necessary requirements for positive regulatory competence. The existence of silent AR occupied regions, however indicates that AR occupancy does not necessarily lead to the recruitment of histone acetylases and subsequent nearby gene expression. Therefore, the rate-limiting step for AR-mediated gene expression is not occupancy *per se*. Silent occupied regions may act as a reservoir of bound AR, possibly poised to mediate gene expression as conditions change. Such conditions may include chromatin modifications and/or the recruitment of coregulators, since most of the AR occupied regions were also co-occupied by other TFs, which in several cases acted as coregulators of gene expression. We conclude that the diversity of site-specific functions of the AR points to differential regulation of gene expression by the same transcription factor related to chromatin structure and the presence of coregulators.

## Materials and Methods

### Cell Culture and Materials

Human prostate cancer C4-2B cells, obtained from ViroMed Laboratories (Minneapolis, MN), were maintained in RPMI 1640 supplemented with 5% (v/v) fetal bovine serum (FBS). Antibodies were anti-AR (N20), anti-HNF-3α (H-120), anti-NFI (H-300), anti-C/EBPβ (C19), anti-GATA2 (H116), normal rabbit IgG (Santa Cruz Biotechnology, Santa Cruz, CA), anti-Oct1 (Abcam Inc., Cambridge, MA) and anti-AcH3-K9/K14 (Upstate Biotechnology Inc., Lake Placid, NY). Pre-designed SMARTpool siRNA reagents against FoxA1, NFI, C/EBPβ, GATA2 and nonspecific siRNA were purchased from Dharmacon (Lafayette, CO). AR siRNA was described previously [24]. TaqMan qPCR probes were obtained from Biosearch Technologies (Novata, CA).

### ChIP-chip

C4-2B cells were cultured in phenol red-free RPMI 1640 supplemented with 5% charcoal/dextrane-stripped FBS (CSS) for 3 days. After 4 h DHT (10 nM) treatment, ChIP was conducted as described previously [23] except that no salmon sperm DNA was used as blocking reagent. The immunoprecipitated DNA and un-enriched input DNA were treated with RNase A and purified using the QIAquick PCR purification kit (Qiagen, Valencia, CA). The purified DNA was blunt-ended using T4 polymerase (New England BioLabs, Ipswich, MA), ligated to the linkers (oJW102, 5′-GCGGTGACCCGGGAGATCTGAATTC-3′, and oJW103, 5′-GAATTCAGATC-3′), and amplified by ligation-mediated PCR (LM-PCR). NimbleGen Systems (Madison, WI) labeled amplified input DNA and ChIP DNA with Cy3 and Cy5, respectively, and hybridized to genomic tilling arrays HG17Tilling Set 35.

### Peak calling

Normalized log-ratios for each replicate were obtained from NimbleGen and processed independently. Moving averages *M_i_* were determined for 600-bp windows centered on each probe *i*, for all windows containing three or more probes. Statistically significant windows were determined by comparing actual moving average values to a null distribution obtained by permutation sampling as follows. For each actual moving average *M_i_* containing *j* probes, we computed a null moving average M^*^_i_ by randomly sampling *j* log-ratio values from the entire dataset. This was performed 10,000 times to generate a distribution of permuted moving averages. A normal QQ-plot (Figure S5) indicated that this distribution was approximately normal except for outliers in the right tail, so we estimated mean (*û*) and variance (*ô^2^*) parameters and performed a one-sided normal test to calculate individual window p-values based on this null distribution. To accommodate the large number of statistical tests, we used the method of Benjamini et. al. to control the False Discovery Rate (FDR) at an FDR (α) level of 0.005. Regions with at least four consecutive probes were considered a peak, then peaks were extended until the first insignificant probe was encountered, and overlapping peaks were merged. For AcH3 occupied regions, final peaks were those that were called in both of the replicate experiments. For AR occupied regions (ARORs), L1 peaks were defined as those present in all three replicate experiments, while L2 peaks were those present in two of three.

### Genomic properties of AcH3 peaks and ARORs (Fig 1D)

Distance to TSSs and genomic contexts were determined relative to transcript annotations in the “Known Genes” track of the UCSC Genome Browser. 6,200 randomized ARORs were generated by picking random regions from chromosomes 19 and 20 with properties matched to the actual L1 ARORs – they had the same relative frequencies on chromosome 19 vs. 20, were matched for size, and were required to have no more than one missing probe. 95% confidence intervals are defined as the TSS distances of the upper 2.5% and the lower 2.5% of the randomized ARORs.

### Conventional ChIP Assay

ChIP analyses on LNCaP or C4-2B cells were performed as described previously [23]. DNA samples from ChIP preparations were analyzed by qPCR using TaqMan PCR Master Mix (Applied Biosystems, Branchburg, NJ). The primers and probes are listed in Table S4.

### Expression Microarray

Hormone-depleted C4-2B cells were treated with DHT (10 nM) or an equal volume of ethanol for 16 hr. Total RNA from three biological replicates was extracted using Aurum Total RNA Kit (Bio-Rad, Hercules, CA) and hybridized to Human-6 v2 Expression BeadChIP (Illumina, San Diego, CA) at the USC/Norris Cancer Center Core Facility. To determine differentially expressed genes, we performed a two-tailed t-test (equal variance) across three DHT− replicates and three DHT+ replicates for all 46,713 array features having a valid measurement for all 6 replicates. To determine an empirical null distribution, we generated 1,000 permutations of the data matrix by randomly shuffling the three DHT− and three DHT+ assignments for each array feature (yielding roughly 47 million randomized features). An adjusted p-value was then determined for each feature. These p-values were converted to Expectation values (E-values) by multiplying by the number of features (Table S2). A significance cutoff of E< = 150 was chosen because it minimized the FDR (at about 0.1) and included all well-studied AR targets in prostate cancer cells. For AROR comparisons, we only considered the 1,232 RefSeq transcripts covered by the chromosome 19 & 20 regions on the NimbleGen tiling array, and set a significance cutoff of E< = 5, corresponding to the E-value chosen for the full genome, and also occurring at the minimum achievable False Discovery Rate (FDR) of 0.1. This cutoff also included all features with fold changes of 1.5 or greater, and included all well-studied AR targets in prostate cells. An AROR was considered “adjacent” to a RefSeq feature if it was within the annotated gene or one of its introns, or if it was upstream or downstream of the gene with no other intervening genes.

### Inter-AROR distances (Figure 3C & D)

Distances were calculated between each pair of AcH3−ARORs (3C) and each pair of AcH3+ARORs (3D). Chromosome and size-matched randomized ARORs were generated as described above. For each trial, we generated sets with the same number of ARORs as the experimentally determined set. For 95% confidence interval, we took the upper 2.5% and the lower 2.5% of distances for 10,000 independent trials.

### Construction of Plasmids

The AROR sequences (an average of 500 bp fragment surrounding the AROR peak center) were PCR amplified from C4-2B genomic DNA and subcloned upstream of a thymidine kinase (TK) minimal promoter-luciferase vector [from Dr Axel Schönthal (USC)] in both directions. The primers for subcloning are listed in Table S5.

### Luciferase Assay and DHT responsiveness

C4-2B (1×10^5^ cells/well) were plated in 12-well plates and grown in phenol red-free RPMI 1640 containing 5% CSS for 2 days. Cells were then transfected with AROR containing TK-luciferase reporter plasmids using Lipofectamine LTX Reagent (Invitrogen Corp., Carlsbad, CA) according to the manufacturer's protocol. After transfection, cells were treated with DHT (10 nM) or ethanol vehicle for 24 h. Luciferase activities were measured as previously described [23]. For each construct, two independent transformants were measured in the DHT− condition and two in the DHT+ condition. Log intensities were used to perform a one sided t-test (assuming equal variance) to determine a p-value. We used log intensities because inter-sample variance was correlated with raw intensity scores but not with log intensity scores. The same procedure was performed for each construct in the reverse orientation, and the minimum of the forward and reverse p-values was recorded. In some cases, this procedure was repeated several times for the same construct, in which case we took the median p-value. For Figure 4A, this p-value is plotted against the intensity fold changes, which were averaged using the geometric mean.

### Motif searches

BioProspector, MDScan, and Weeder were used to identify motifs *de novo* by comparing the 60 L1 ARORs (we excluded A002 which has 14 tandem AREs, and A059 which could not be cloned) to 6,200 size-matched controls. Each was run to search for motifs of various lengths (6–22 for MDScan and BioProspector, 6–12 for Weeder). The “-T 20” command line parameter was used for BioProspector, and “-t 30” was used for MDScan. Because these *de novo* search programs produce a large number of highly similar versions of the same motif (408 for MDscan and BioProspector, 125 for Weeder), we performed a clustering technique to collapse them to a relatively non-redundant set. We examined each possible pair of motifs *A* and *B*, and measured the Jaccard distance between the two motifs, which is defined as 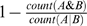, where *count(A&B)* is the number of overlapping binding sites between *A* and *B*, and *count(A|B)* is the total number of binding sites predicted for either motif. Motifs were then clustered using linear hierarchical clustering (average linkage), and motifs with an average Jaccard distance of 0.1 or less were grouped together. The motif with the lowest average distance to all other motifs in the cluster was recorded as the prototype and used for all subsequent analysis. Weeder produced 23 clusters, and BioProspector and MDScan results were combined to yield 22. To this we added 2 versions of the ARE motif from JASPAR/ConSite, and 5 motifs identified by CEAS (ARE, HNF3α, NFI, C/EBP, and GR) [28] to yield a total of 52 enriched motifs (Table S3).

### Motif enrichment in DHT-responsive ARORs (Figure 4B)

For each of the constructs with luciferase data, we counted the number of predicted binding sites within the construct for each of the 52 motifs enriched in ARORs (4B, upper), and separately counted the number of predicted sites for each of the 1,326 unique motif pairs (4B, lower). For each motif or motif pair, we generated an enrichment significance score by performing a one-sided non-parametric t-test (assuming equal variance) between the binding site counts in the 18 strongly induced ARORs vs. the 19 non-responsive ARORs. Adjusted p-values were determined by randomly permuting the non-responsive and responsive labels 1,000 times for each motif, and determining an empirical distribution of t-test scores, which was used to calibrate the actual t-test scores.

### siRNA Transfection

C4-2B (1×10^5^ cells/well) were plated in 6-well plates and grown in phenol red-free RPMI 1640 containing 5% CSS for 2 days. Cells were transfected with the siRNA duplexes as indicated at a final concentration of 100 nM using Oligofectamine Reagent (Invitrogen) according to the manufacturer's instructions. After transfection, cells were grown in phenol red-free RPMI 1640 containing 5% CSS for 48 hr and then treated with DHT (10 nM) or ethanol vehicle for additional 18 hr. Total RNA extraction and protein extraction were conducted respectively for further assessment.

### qRT-PCR

After the indicated treatments, total RNA from C4-2B cells was extracted using Aurum Total RNA Kit (Bio-Rad, Hercules, CA). cDNA was prepared using iScript cDNA Synthesis Kit (Bio-Rad), and qPCR was conducted using TaqMan PCR Master Mix or SYBR Green PCR Master Mix (Applied Biosystems, Branchburg, NJ). The primers and probes are listed in Table S6. Triplicate PCR reactions were conducted. GAPDH mRNA expression was analyzed for each sample in parallel.

### Immunoblotting

Immunoblotting were performed as previously described using the indicated antibodies [50], [51].

## Supporting Information

Figure S1 AROR distribution in Chromosomes 21 & 22 of LNCaP cells. Data from Wang et al [3].
(0.09 MB TIF)

Figure S2 Conservation of AR ChIP and AcH3 ChIP non-coding regions. Cumulative distribution plots of 28-way phastCons conservation scores (UCSC phastCons28way) are plotted for the non-exonic ARORs, Estrogen Receptor Occupied Regions (ERORs) from [2], and known enhancer elements taken from [52]. “Random” ARORs are the non-exonic subset of the size-matched randomized chromosome 19 & 20 regions described in Figure 1D. ERORs and ARORs appear to be only slightly more conserved than the non-coding genomic background.
(7.99 MB TIF)

Figure S3 Hormone-dependent gene expression in C4-2B cells. Illumina expression arrays were used to measure expression levels of 46,713 transcripts in three replicates before and three replicates after DHT exposure in C4-2B cells. The student's t-test was used to determine statistical significance, and p-values were adjusted based on random permutations of the full dataset. This volcano plot shows the E-value (number of transcripts at the given p-value expected by chance) plotted against the mean fold change. 552 transcripts up-regulated at the E = 150 level (permutation-adjusted p-value = 0.004), along with 416 transcripts down-regulated (permutation-adjusted p-value = 0.003), are shown in the upper two quadrants, and of the well-studied DHT-responsive genes in prostate cancer are labeled.
(2.42 MB TIF)

Figure S4 Predicted ARE and coregulator binding sites in L1 ARORs. Binding sites for the ARE and 3 coregulator motifs are shown for the AROR constructs either highly induced (upper left), weakly induced (lower left), unchanged (upper right), and repressed (lower right) in our luciferase reporter activity assays. The strongly induced ARORs had a total of 70 sites in 18 ARORs (3.9 sites per AROR), while the weakly induced had 35 sites in 21 ARORs (1.7 sites per AROR) and the unchanged had 20 sites in 19 ARORs (1.6 sites per AROR).
(40.51 MB TIF)

Figure S5 Normality of permuted tiling array moving averages. Individual log-ratios from one of the AR-ChIP NimbleGen arrays were permuted into “randomized” moving averages (the number of probes per window were matched to actual 600-bp windows on the array). 10,000 randomized moving averages were sampled and plotted against the standard normal. The results show that they largely follow a normal distribution. The deviating right tail comes from enriched probes, but represent only a small fraction of all values and do not effect the statistical testing described above which aims to reject against the background distribution, i.e. the bulk of moving average scores.
(0.88 MB TIF)

Figure S6 Occupancy of AR and coregulators in LNCaP and C4-2B cells. LNCaP and C4-2B cells were cultured in hormone-depleted medium for 3 days and then treated with 10 nM DHT or ethanol vehicle for 4 h. Conventional site-specific ChIP assays were performed with indicated antibodies. The values are presented as percentage of input.
(3.54 MB TIF)

Figure S7 Occupancy of AR and coregulators at PSA and TGM loci. C4-2B cells were cultured in hormone-depleted medium for 3 days and then treated with 10 nM DHT or ethanol vehicle for 4 h. Conventional site-specific ChIP assays were performed with indicated antibodies. PSA and TGM ARORs were re-plotted from Figure 5. The values are presented as percentage of input.
(51.64 MB TIF)

Table S1 189 L1/L2 ARORs and 1,189 AcH3 occupied regions
(0.60 MB XLS)

Table S2 Hormone-dependent gene expression in C4-2B cells
(4.91 MB XLS)

Table S3 Sequence motifs enriched in ARORs
(0.08 MB XLS)

Table S4 ChIP-PCR, probes and primers
(0.04 MB XLS)

Table S5 Luciferase reporter constructs, probes and primers
(0.03 MB XLS)

Table S6 Quantitative PCR, probes and primers
(0.03 MB XLS)

Table S7 IlluminaGX.txt.gz. Illumina microarray raw expression data of C4-2B cells. Text file.
(3.08 MB GZ)
