# Supplementary figures and images for: Genomic Androgen Receptor-Occupied Regions with Different Functions, Defined by Histone Acetylation, Coregulators and Transcriptional Capacity
Source: PLoS One. 2008 Nov 10;3(11):e3645. doi: 10.1371/journal.pone.0003645 (PMC2577007; doi:10.1371/journal.pone.0003645)

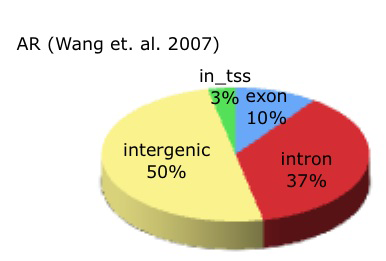

Supplement: Figure S1 — AROR distribution in Chromosomes 21 & 22 of LNCaP cells. Data from Wang et al [3]. (0.09 MB TIF) [file pone.0003645.s001.tif]

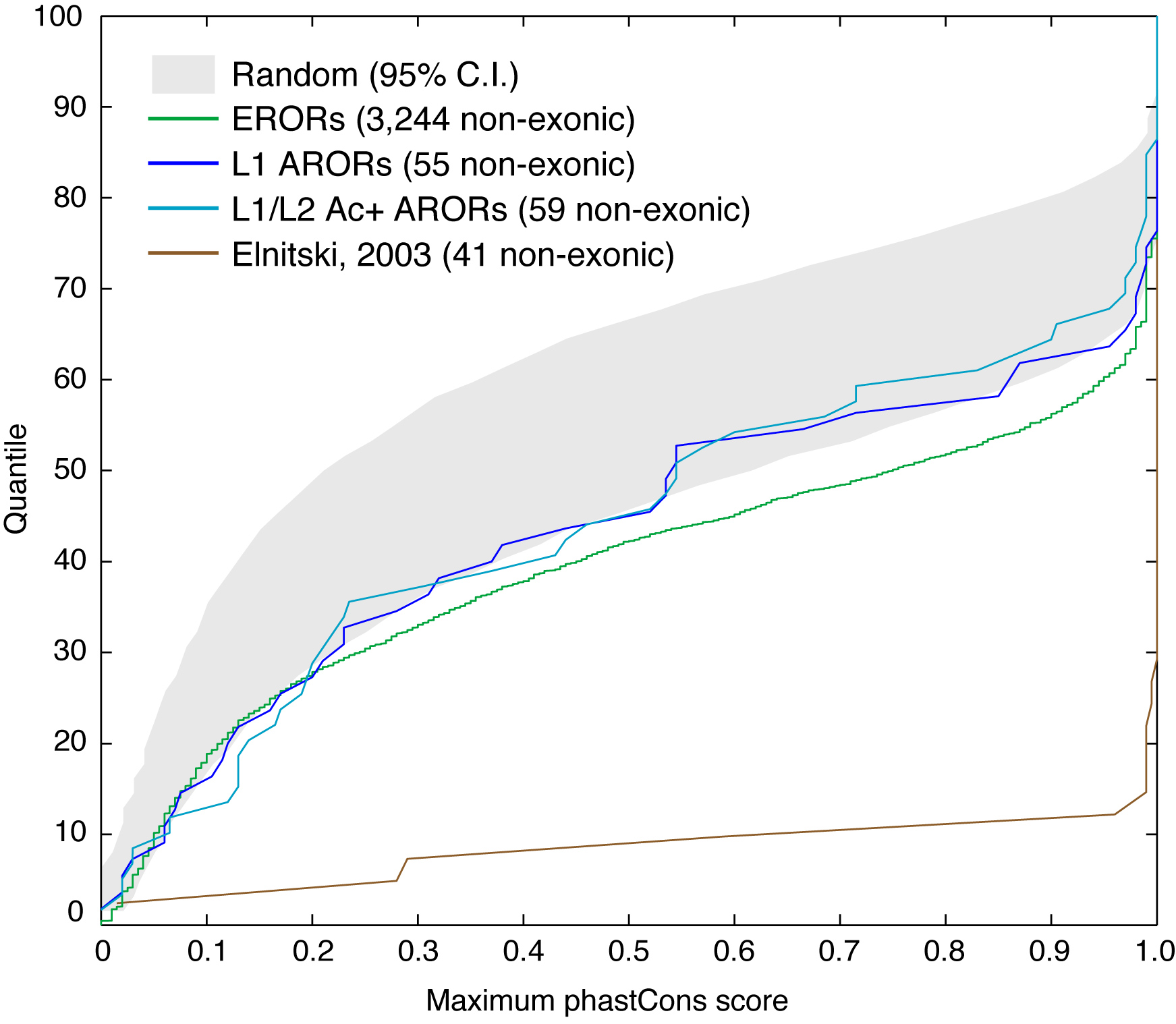

Supplement: Figure S2 — Conservation of AR ChIP and AcH3 ChIP non-coding regions. Cumulative distribution plots of 28-way phastCons conservation scores (UCSC phastCons28way) are plotted for the non-exonic ARORs, Estrogen Receptor Occupied Regions (ERORs) from [2], and known enhancer elements taken from [52]. “Random” ARORs are the non-exonic subset of the size-matched randomized chromosome 19 & 20 regions described in Figure 1D. ERORs and ARORs appear to be only slightly more conserved than the non-coding genomic background. (7.99 MB TIF) [file pone.0003645.s002.tif]

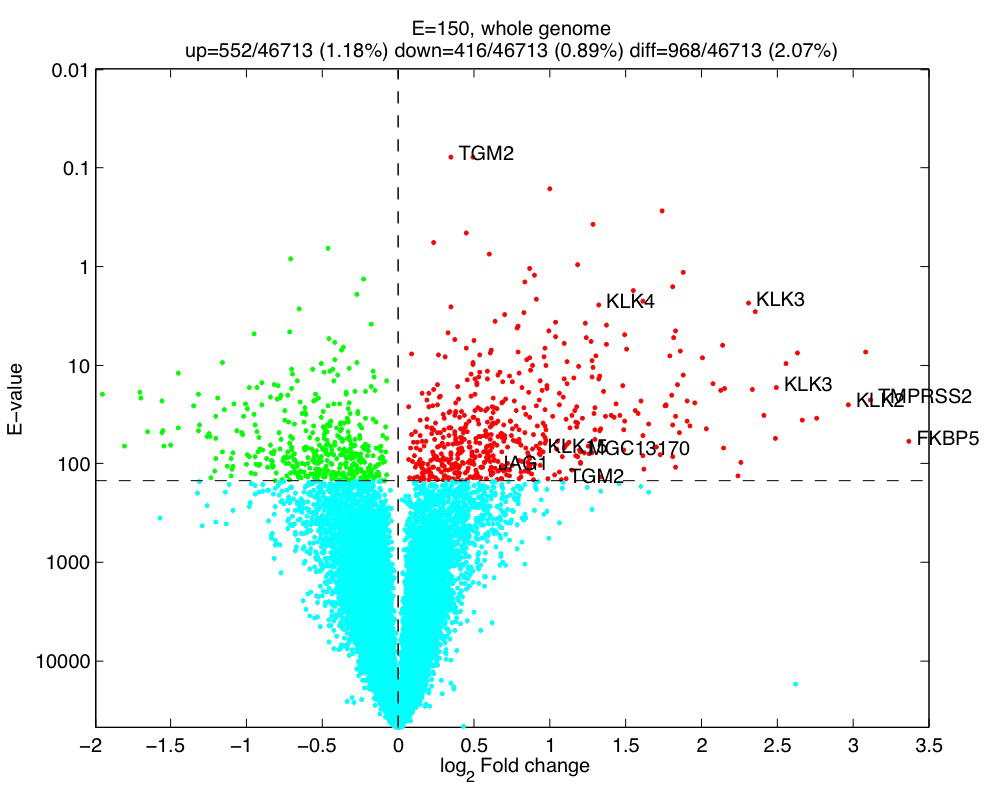

Supplement: Figure S3 — Hormone-dependent gene expression in C4-2B cells. Illumina expression arrays were used to measure expression levels of 46,713 transcripts in three replicates before and three replicates after DHT exposure in C4-2B cells. The student's t-test was used to determine statistical significance, and p-values were adjusted based on random permutations of the full dataset. This volcano plot shows the E-value (number of transcripts at the given p-value expected by chance) plotted against the mean fold change. 552 transcripts up-regulated at the E = 150 level (permutation-adjusted p-value = 0.004), along with 416 transcripts down-regulated (permutation-adjusted p-value = 0.003), are shown in the upper two quadrants, and of the well-studied DHT-responsive genes in prostate cancer are labeled. (2.42 MB TIF) [file pone.0003645.s003.tif]

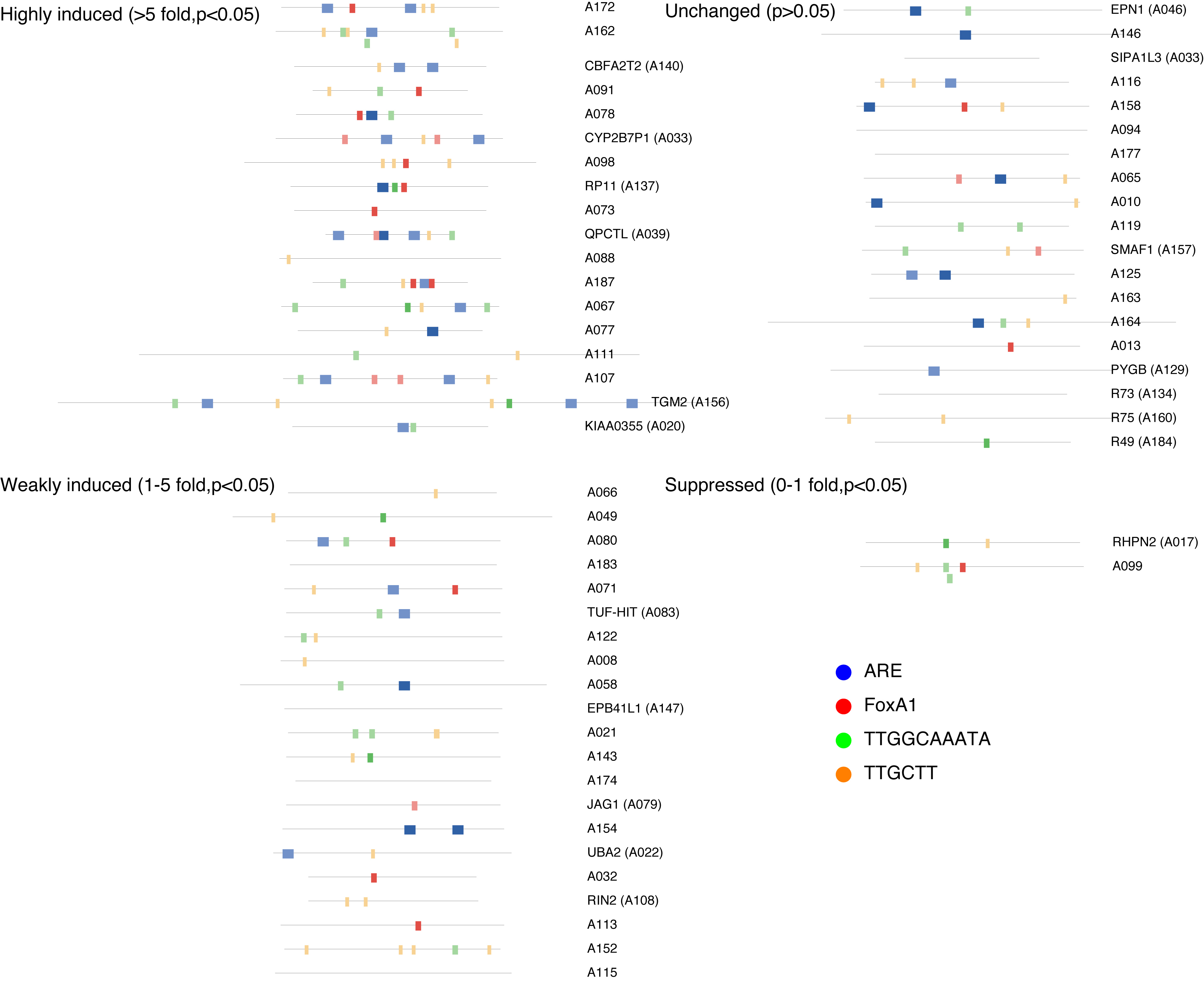

Supplement: Figure S4 — Predicted ARE and coregulator binding sites in L1 ARORs. Binding sites for the ARE and 3 coregulator motifs are shown for the AROR constructs either highly induced (upper left), weakly induced (lower left), unchanged (upper right), and repressed (lower right) in our luciferase reporter activity assays. The strongly induced ARORs had a total of 70 sites in 18 ARORs (3.9 sites per AROR), while the weakly induced had 35 sites in 21 ARORs (1.7 sites per AROR) and the unchanged had 20 sites in 19 ARORs (1.6 sites per AROR). (40.51 MB TIF) [file pone.0003645.s004.tif]

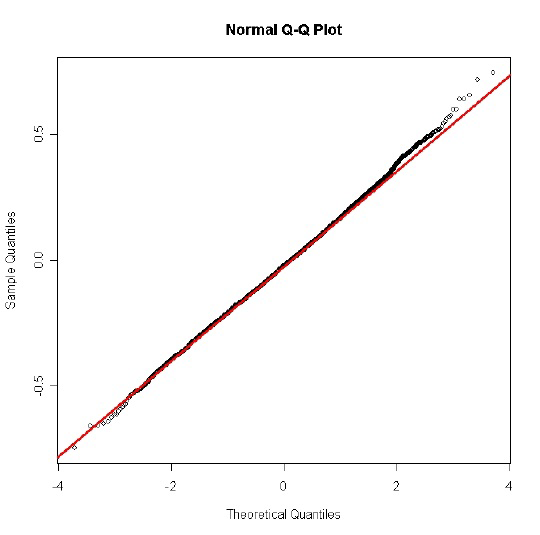

Supplement: Figure S5 — Normality of permuted tiling array moving averages. Individual log-ratios from one of the AR-ChIP NimbleGen arrays were permuted into “randomized” moving averages (the number of probes per window were matched to actual 600-bp windows on the array). 10,000 randomized moving averages were sampled and plotted against the standard normal. The results show that they largely follow a normal distribution. The deviating right tail comes from enriched probes, but represent only a small fraction of all values and do not effect the statistical testing described above which aims to reject against the background distribution, i.e. the bulk of moving average scores. (0.88 MB TIF) [file pone.0003645.s005.tif]

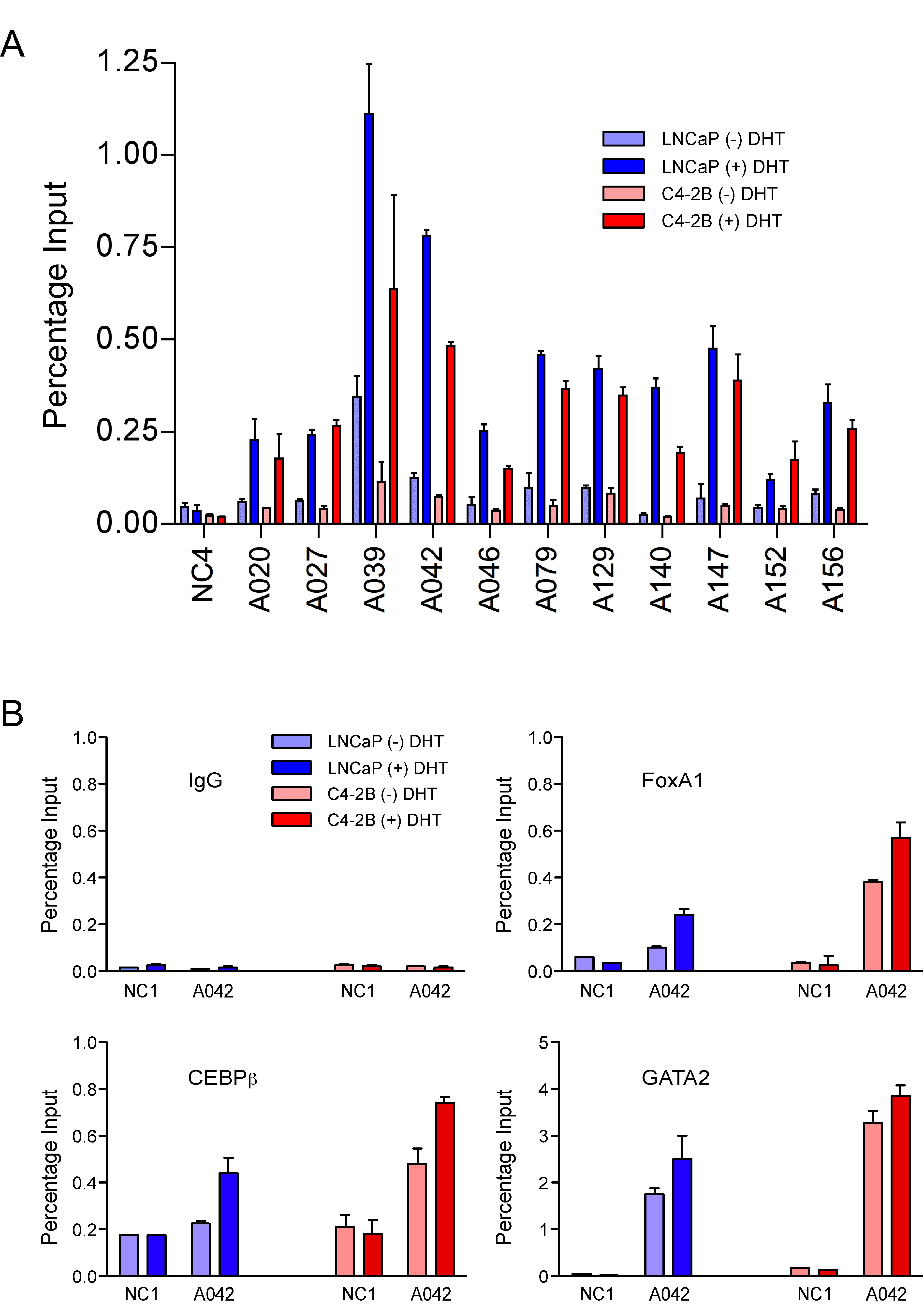

Supplement: Figure S6 — Occupancy of AR and coregulators in LNCaP and C4-2B cells. LNCaP and C4-2B cells were cultured in hormone-depleted medium for 3 days and then treated with 10 nM DHT or ethanol vehicle for 4 h. Conventional site-specific ChIP assays were performed with indicated antibodies. The values are presented as percentage of input. (3.54 MB TIF) [file pone.0003645.s006.tif]

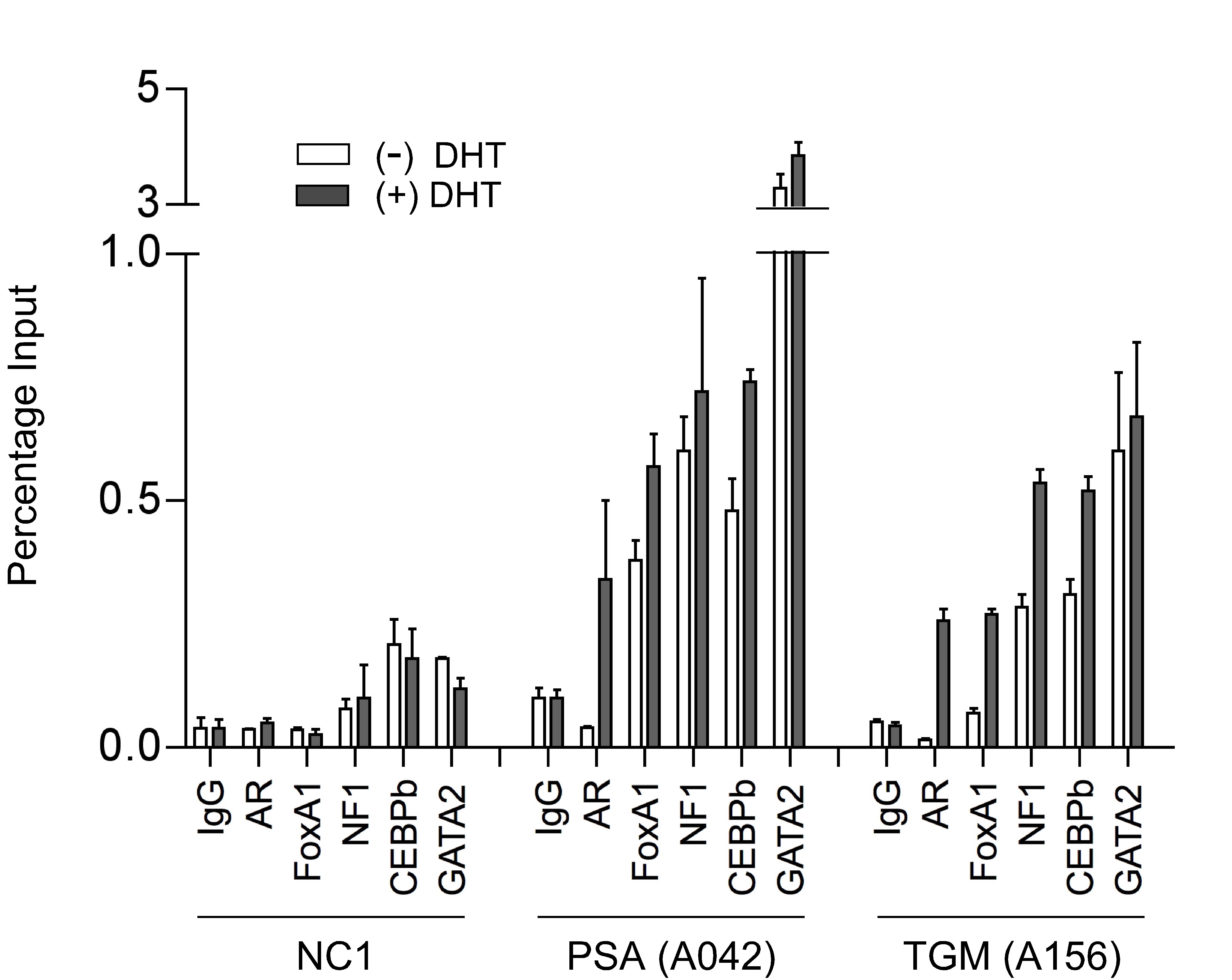

Supplement: Figure S7 — Occupancy of AR and coregulators at PSA and TGM loci. C4-2B cells were cultured in hormone-depleted medium for 3 days and then treated with 10 nM DHT or ethanol vehicle for 4 h. Conventional site-specific ChIP assays were performed with indicated antibodies. PSA and TGM ARORs were re-plotted from Figure 5. The values are presented as percentage of input. (51.64 MB TIF) [file pone.0003645.s007.tif]
